# Simultaneous Analysis and Quality Assurance for Diffusion Tensor Imaging

**DOI:** 10.1371/journal.pone.0061737

**Published:** 2013-04-30

**Authors:** Carolyn B. Lauzon, Andrew J. Asman, Michael L. Esparza, Scott S. Burns, Qiuyun Fan, Yurui Gao, Adam W. Anderson, Nicole Davis, Laurie E. Cutting, Bennett A. Landman

**Affiliations:** 1 Department of Electrical Engineering, Vanderbilt University, Nashville, Tennessee, United States of America; 2 Institute of Imaging Science, Vanderbilt University, Nashville, Tennessee, United States of America; 3 Department of Computer Science, Vanderbilt University, Nashville, Tennessee, United States of America; 4 Department of Radiology and Radiological Sciences, Vanderbilt University, Nashville, Tennessee, United States of America; 5 Department of Biomedical Engineering, Vanderbilt University, Nashville, Tennessee, United States of America; 6 Special Education, Vanderbilt University, Nashville, Tennessee, United States of America; University College London, United Kingdom

## Abstract

Diffusion tensor imaging (DTI) enables non-invasive, cyto-architectural mapping of in vivo tissue microarchitecture through voxel-wise mathematical modeling of multiple magnetic resonance imaging (MRI) acquisitions, each differently sensitized to water diffusion. DTI computations are fundamentally estimation processes and are sensitive to noise and artifacts. Despite widespread adoption in the neuroimaging community, maintaining consistent DTI data quality remains challenging given the propensity for patient motion, artifacts associated with fast imaging techniques, and the possibility of hardware changes/failures. Furthermore, the quantity of data acquired per voxel, the non-linear estimation process, and numerous potential use cases complicate traditional visual data inspection approaches. Currently, quality inspection of DTI data has relied on visual inspection and individual processing in DTI analysis software programs (e.g. DTIPrep, DTI-studio). However, recent advances in applied statistical methods have yielded several different metrics to assess noise level, artifact propensity, quality of tensor fit, variance of estimated measures, and bias in estimated measures. To date, these metrics have been largely studied in isolation. Herein, we select complementary metrics for integration into an automatic DTI analysis and quality assurance pipeline. The pipeline completes in 24 hours, stores statistical outputs, and produces a graphical summary quality analysis (QA) report. We assess the utility of this streamlined approach for empirical quality assessment on 608 DTI datasets from pediatric neuroimaging studies. The efficiency and accuracy of quality analysis using the proposed pipeline is compared with quality analysis based on visual inspection. The unified pipeline is found to save a statistically significant amount of time (over 70%) while improving the consistency of QA between a DTI expert and a pool of research associates. Projection of QA metrics to a low dimensional manifold reveal qualitative, but clear, QA-study associations and suggest that automated outlier/anomaly detection would be feasible.

## Introduction

Diffusion tensor imaging (DTI) is a magnetic resonance (MR) imaging technique that provides contrasts uniquely sensitive to intra-voxel tissue microarchitecture on a scale of tens of microns [1]. DTI has transformed MR neuroimaging studies and has found wide-spread applications in non-invasive assessment of white matter microstructure, reconstruction of major fiber bundles, and mapping of in vivo brain connectivity. A DTI experiment can consist of up to 90 or more volumes, be aggressive on hardware particularly gradients, and be susceptible to standard as well as unique artifacts [2]; high data quality is difficult to maintain. However, DTI data quality analysis is exceedingly difficult. With multi-volume datasets, artifacts can present as natural variability across the volumes and quality assurance (QA) by manual inspection becomes time consuming and tedious. Upgrades or hardware changes that impact noise structures may adversely impact statistical compatibility across DTI datasets. Additionally, the processing of DTI data involves mapping data to a logarithmic diffusion model which is well-known to induce bias in measured parameters [3], [4]. Because of these challenges, DTI data quality analysis is usually reserved as a separate step post data collection, reducing the chances of an experimental response to poor data quality. The documented challenges of producing accurate and biophysically meaningful metrics from a DTI experiment suggest DTI is an important target for further QA development.

Important headway has been made towards developing quality analysis methods for DTI. As in other MRI sub-disciplines which have begun evaluating data quality through automatic pipeline methods [5], [6], [7], [8], the DTI field also offers DTI pipeline programs. DTIPrep offers quality assurance primarily through artifact detection [9]. Similarly, DTI processing programs, such as CATNAP [10], and DTI studio [11], offer streamlined processing which can assist and enable the researcher to spend more time on QA. Although existing image DTI processing and artifact detection programs can significantly aide DTI QA evaluation, they cannot address quality concerns that do not manifest as artifacts.

Much quality analysis work has been done outside the realm of artifact detection. These include sophisticated analyses developed to understand noise structures in DTI and to investigate the impact of noise on the quality of measured diffusion properties [9], [12], [13], [14], [15], [16], [17], [18]. To evaluate general hardware performance, phantoms tailored for DTI have been developed [19], [20], [21]. There have also been several recent developments of modern statistical metrics with demonstrated sensitivity to empirical DTI data quality [22], [23], [24], [25]. Although the rapid advancement in DTI quality analysis has produced a scope and scale of publications that is exciting, the statistical advances are difficult for clinical researchers to efficiently incorporate into their own research, and it has remained uncertain if these theoretical advancements would yield practical benefits. Additionally, implementation of any existing method requires manual execution and does not address the potentially crucial time delay between data collection and data quality analysis.

To address the concerns of time delays and limited access to statistical methods for improved QA of DTI data, we present an in situ quality analysis pipeline. Greater access to statistical methods is enabled by the incorporation of multiple statistical metrics from the literature. The metrics evaluate the collected image data (patient motion estimates, voxel outlier detection, noise-sensitive evaluation of fitting errors), the processed tensor parameters (bias estimates, bootstrap standard deviation, boxplot distribution analysis) and are complementary to the existing DTI QA artifact detection software package, DTIPrep. The pipeline also takes advantage of existing software packages for DTI data processing and analysis [26], [27], [28]. To bridge the time gap between data collection and human QA assessment, the pipeline can run automatically post data collection and produces a four page report that graphically summarizes the various pipeline outputs. This report is produced within 24 hours of the DTI experiment and provides a quick and easy overview of QA metrics and the image data.

Researchers can visualize and develop exploratory analysis methods on the stored pipeline outputs, thus granting easier access to robust parameter estimates, statistical analysis outcomes, and an encouragement of new data exploration. All statistical metrics have been previously demonstrated useful for DTI QA and providing researchers easier access to these methods is a clear benefit of the pipeline. What remains to be evaluated are two important pieces of the pipeline; (i) the effectiveness of the chosen graphical visualization for aiding human QA and (ii) demonstrating an advantage, if any, of unifying the statistical pieces and offering at least one possible avenues for data exploration that combines these pieces.

Herein we present the pipeline, the QA report, an evaluation of the QA report, and an analysis of the pooled statistical outputs. To test the pipeline, the program is run retrospectively on 608 DTI datasets from various studies. The effectiveness of the QA report is tested using a multiple-choice four question QA rubric designed in collaboration with local DTI clinical researchers. Rubric answers from eight novices evaluating (a) image data only or (b) the QA report only are compared to experienced evaluation of 50 random DTI datasets. The QA report is rated on its ability to enable novice agreement with expert evaluation and on the time saved. To evaluate if the unified statistical outputs quantify key characteristics of the DTI data, each of the DTI datasets is summarized by a 112 element vector consisting of QA outputs. Principal component analysis (PCA) data reduction to two dimensions is used to investigate data clusters and evaluate if the statistics collaboratively report on defining data characteristics that could be used for outlier detection.

## Theory

The QA pipeline incorporates distinct statistical metrics and processing modules. Here, we provide background on important pipeline segments. Segments that are part of software packages (CAMINO, FSL) are not included in the theory background. The pieces described below include a segmentation method, a modified goodness of fit evaluation (pixel chi-squared), estimates of FA standard deviation, estimates of FA bias, and estimated power curves. Except for a minor adaptation to the pixel chi-squared, all sections (2.1 – 2.6) described below explain methods developed outside the context of this manuscript.

### 2.1 DTI Diffusion Model

To clarify use of terms, a brief background on DTI is provided. Several detailed reviews have been published [1], [29], [30]. Briefly, DTI measures the three-dimensional diffusion of water in vivo which is described mathematically by a 3×3 diffusion tensor (**D**). A DTI experiment consists of a series of diffusion weighted images (DWI) that are each sensitized to diffusion along the direction of an applied gradient, indexed by *j* = *1,2 ,…J* (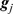). The DWIs are divided by a non-diffusion weighted image, b_o_, yielding *J* normalized images, *S_j_*. The relationship between the data ***S*** and the diffusion tensor is described by the Stejskal-Tanner relation,



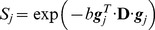
(1)


As is common, *b* is the scalar b-value describing the magnitude of diffusion weighting as determined by experimental protocols. The collection of gradient directions, (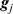), is denoted the ‘gradient table’, and varies for different DTI protocols. Several important summary metrics are derived from the Eigenvalues of **D**, 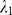, 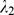, and 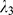, and Eigenvectors of **D**, ***e1***, ***e2*** and ***e3***. Here ***e1*** corresponds to the largest Eigenvalue 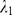 while ***e3*** corresponds to the smallest, 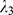. Two important summary scalar metrics are mean diffusivity (MD) which measures the average diffusion across all directions and fractional anisotropy (FA) which measures the level of anisotropic diffusion (Eq.2).
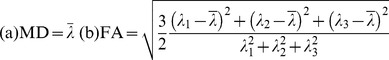
(2)


### 2.2 Multi-Atlas Segmentation

We use multi-atlas segmentation to automatically segment previously unseen b_o_ volumes of DTI datasets. In the pipeline, the labeled b_o_ image is used for regional quality analysis of DTI outputs, MD and FA, as well as for automatic noise estimation (described in Methods). Multi-atlas segmentation represents a highly robust and fully automated class of techniques for segmenting a previously unseen context (target) using an existing labeled dataset (atlases) [31], [32]. In general, multi-atlas segmentation is performed through two successive steps. First, the atlases, consisting of both image intensities and labels, are transformed to the target coordinate system through a deformable image registration [33], [34], [35]. Second, the voxel-wise label conflicts are resolved using label fusion [36], [37], [38] to form a final estimate of the underlying target segmentation.

### 2.3 Modified Goodness of Fit Assessment

To quantify how well the image data fits the diffusion model (Eq. 1), we turn to a ‘goodness of fit’ measure. Traditional statistics uses the chi-squared metric to measure goodness of fit. Interestingly, a modified chi-squared metric, the pixel chi-squared, 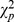, has been adapted and tested specifically for DTI. Unlike the traditional chi-squared, the pixel chi-squared has the property of being sensitive to image noise [16]. To explain the difference we examine the mathematical expressions for the two metrics. In Eq. 3, the pixel chi-squared, 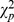 , is compared to the traditional chi-squared, 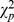.
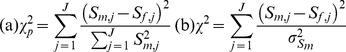
(3)


For both metrics the fitted diffusion tensor from measured normalized image data, ***S_m_***, is projected back through the diffusion model (Eq. 1) to create fitted normalized image data, ***S_f_***. Error is then defined ***S_m_*** **–** ***S_f_***. The pixel chi-squared normalizes the errors based upon the signal intensities of the *S_m,j_*, while the traditional chi-squared normalizes based upon their variance, 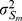. Note the traditional chi-squared maps both poor fitting ‘bad data’ and low noise ‘good data’ to large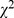; [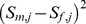
>> 0, 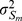
<< 0]. The modification by Papadakis et. al enabled the mapping of both poor fitting ‘bad data’ *and* high noise ‘bad data’ to large 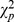 while well fitting ‘good data’ and low noise ‘good data’ is mapped to small 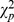. Papadakis et al demonstrated that 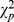 values cluster in two groups, a signal region centered just above zero, and a ‘noise’ region centered near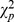 ∼ 0.2. These two regions are referred to as ‘signal lobe’ and ‘noise lobe’.

Originally 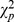 was proposed as a noise filtering method, but herein we adapt the metric for QA. Instead of summing the fitting residuals of *S_mj_* across all *j*, 1< = *j* < = *J*, at a single spatial location, we sum across all spatial locations, *k = 1,2, … K* within an axial-slice from a single normalized DWI. To maintain intensity compatibility across slices with different number of spatial locations and to maintain compatibility with the original 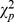 the sum is multiplied by an additional normalizing factor, *J/K* (Eq. 4).
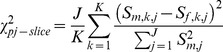
(4)


To be clear, Eq. 3 produces one 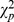 value per spatial location while Eq. 4 produces one 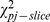 value per slice per normalized DWI. The new 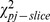 can then be used to summarily evaluate each axial slice of the normalized DWI based upon noise and the fitting errors.

### 2.4 Estimates of FA Standard Deviation

Wild-bootstrap and bootstrap methods can be used to predict the experimental variance of FA, 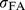, given only a single DTI dataset. The wild-bootstrap adapted for DTI has recently been demonstrated to produce good estimates of 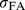 [39], and confidence intervals based upon bootstrap estimates of 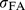 have been demonstrated to be sensitive to DTI data quality [23]. The premise of bootstrap is that the empirical standard deviation of FA that *would* be measured *if* repeated datasets were collected can be estimated through Monte-Carlo simulation of datasets with similar statistical properties as the empirically measured dataset.

There are several possible approaches for implementing a wild-bootstrap, and we present the bootstrap approach through the method used herein. For a fixed spatial location the errors are, ε*_j = _*|*S_m,j_* – *S_f,j_* |. For each Monte-Carlo simulation, the *J* errors are shuffled and given a random sign (+ or -), **±** ***ε*** *_shuffle_*. The use of the random sign is what makes this a ‘wild’ bootstrap method. Wild-bootstrap data, ***S_bs_***_**,**_, is synthesized by adding the shuffled errors to the fitted data: ***S_bs_*** *=* ***S_f_*** *+*( **±** ***ε*** *_shuffle_* ).This process is repeated and each ***S_bs_*** is considered a sampling from a population created to be statistically similar to the population that would result if data were empirically re-measured. Each ***S_bs_*** is fit to the diffusion model (Eq. 1) and a population of FA values is created and 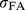 can be estimated.

### 2.5 Estimates of FA Bias

FA estimates are well-known to contain bias [4], [13], [15]. Bias contributes to error in measured FA values and corrupts statistical inferences by shifting the expected FA, E(FA_measured_), away from the true FA value.
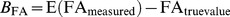
(5)


SIMulation Extrapolation (SIMEX) is a modern statistical method for estimating bias in parameter outputs from empirically fitted mathematical models [40], [41]. SIMEX has very recently been demonstrated to produce accurate estimates of bias in empirical FA values [24]. SIMEX measures bias in FA by first observing the trend in the expected FA value when *additional* synthetic noise of variance *ω* is added to the observed diffusion weighted images, ***DWI_m_***, and b_o_. In the case of DTI, a stacked Rician noise model [42] is used to further corrupt the ***DWI_m_*** to form a noisy dataset ***DWI_m_***_*ω*_. Let the standard deviation of imaging noise in image ***DWI_m_*** be described by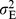, then the elements of the corrupted data set ***DWI_mω_*** are Rician distributed,
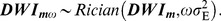
(6)


Similarly for b_o_, 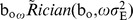. By varying *ω*, the trend in the expected value of FA with additional noise can be modeled. An approximation function (polynomial order 2 in this case) is fit to the trend and then extrapolated to zero-noise (which occurs at *ω* = −1 since ***DWI*** *_m_* has variance 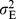 leading ***DWI_m_***_*ω*_ to have total variance, 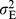 + *ω* 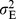). The difference between the measured FA value and the extrapolated FA value represents the estimate of bias.

### 2.6 Power Calculations

Power analyses are included in the pipeline for two key reasons (i) power is a ubiquitous form of statistical evaluation that is accessible and important to clinical researchers, and (ii) power is sensitive to data quality. Power evaluations traditionally require a minimum sample size *n = *3, but bootstrap estimates of 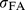
enable the statistically tractable question to be asked of a *single* dataset: ‘If all other *n* datasets in my study were collected with the standard deviation observed in this dataset, 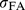,what would be the power of a two-sided *t*-test at an effect size of ES?’ SIMEX also enables incorporation of bias into the power calculation as has been previously demonstrated in the context of DTI [43]. Bias (*B*) is known to impact the power of hypothesis tests and because FA is well known to be biased, it is important to consider bias when evaluating power of FA estimates. Briefly, without bias, power is minimal when the effect size is zero, e.g., there is the smallest chance of detecting a difference when there is none. However, because bias shifts the expectation value of the observed difference away from the true value (Eq. 5) , bias also shifts the power curve minimum away from zero to the new minimum ES = -*B*.

The impact of bias on the power of a two-sided t-test can be explicitly written. Consider two groups with sample size *n*, common estimated standard deviation *s*, and a difference in bias of Δ*B*. Letting T represent the cumulative distribution function for the Student’s *t* distribution with degrees of freedom 2*n*-2, and *t_y_* represents the inverse of T at point *y*, then the power of a two-sided *t*-test between these two groups is written,
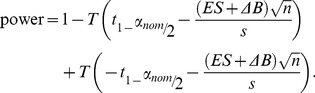
(7)


The *α*_nom_ is the specified false error rate (typically *α*_nom_ = 0.05). Note that when Δ*B* = 0, Eq. 7 reduces to the standard power equation for a two-sided *t*-test.

Although SIMEX estimates of *B*_FA_ enable incorporation of bias into the pipeline power analysis, it is the *difference* in bias (Δ*B*) between groups that determines the impact of bias (Eq. 7). To frame a statistically tractable question without access to a bias estimate for the comparison group, a worst-case scenario approach is used. The question is asked: ‘If all other *n* datasets in my study were collected with the standard deviation observed in this dataset, *s* = 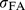, and the *difference* in bias between groups equals my observed bias, (e.g., the comparison group contains no bias and Δ*B = B*_FA_), what would be the power of a two-sided *t*-test to detect an effect size of ES? Letting Δ*B = B*_FA_ is considered the worst case scenario because FA bias tends to correlate with anatomy [4]and the true difference in bias between groups will most likely be less than the bias of either group alone.

## Methods

### 3.1 DTI Data

**Figure 1:**
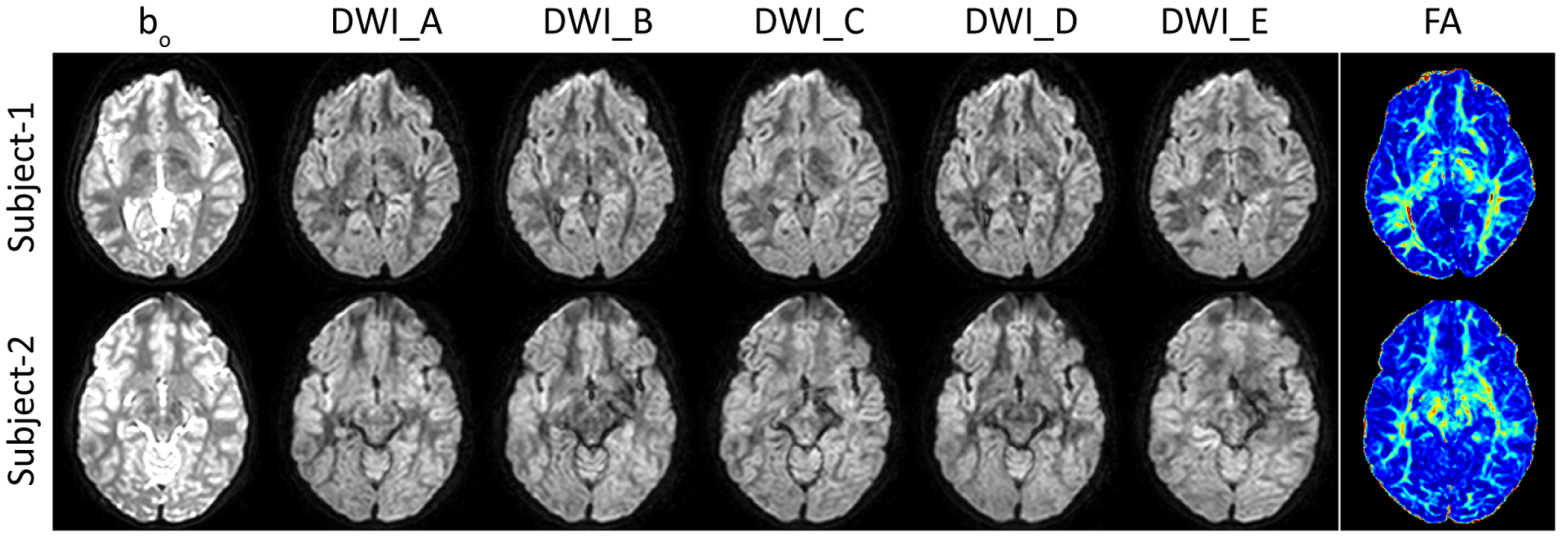
Example input and output data for two subjects from Study II. The DWIs were randomly chosen by a computer but are from the same gradient for each subject. DWIs and b_o_ are shown at different intensity scales for viewing purposes. The FA color scale ranges from [blue-red] and colors map to FA values [0 1]. Inter-subject registration was not performed and the axial slices are from approximate matching locations. The FA map of subject 2 contains an anomalous bright region in the middle-right mid-brain/hemisphere (patient right is image right). This bright region is not clearly traced to DWI artifacts, but upon comparison to a re-scan of the same individual is associated with flow artifacts rather than pathology.

Two primary groups of data are used in this study. The first group consists of 608 DTI datasets that were submitted to the pipeline for analysis. The datasets consisted of 607 datasets from 8 different studies belonging to the same principal investigator, and one study from a different principal investigator. Study procedures were approved by the Institutional Review Boards (IRB) at Johns Hopkins University and Vanderbilt University. Participants were recruited in Baltimore, MD and Nashville, TN. Prior to enrollment in the study all participants, including control participants, were screened using a scripted evaluation tool administered by trained research staff over the telephone. Individuals with brain injury, other physical disabilities, severe emotional problems, uncorrected sensory disorders, or an IQ ≤ 70, all of which may interfere with the specificity of the brain activation patterns, were excluded during recruitment. In addition, children were screened for claustrophobia and possible contra-indicators such as dental braces. No individual who was defined as having limited proficiency in English participated in the imaging study. No restriction was made for gender, ethnicity, or socioeconomic status. Prior to all research procedures, written informed consent was obtained from all adults and children’s guardians. Written assent was obtained from the children. Herein, we access all data retrospectively in anonymous form as proscribed by the overseeing IRB. All DTI data were collected using echo planar imaging (EPI) with an 8-channel head coil on one of four Philips 3T systems. Collection details for the nine studies are listed in **Table 1**. Sub-samples of input and output data from two of the datasets are presented in **Figure 1**. The figure also highlights some important yet difficult aspects of quality analysis in DTI.

**Table 1:** DTI Data.

Study	# Data^2^	# G^3^	b-value (s/mm^2^)	slices^4^	FOV (mm^3^)^1^	Voxel size (mm^3^)^1^
**I**	34(32)	32	700	60	212×212×132	0.8×0.8×2.2
**II**	183(183)	32	700	60	212×212×132	0.8×0.8×2.2
**III^5^**	136(111)	32	700	60, 65, 70	212×212×(132–154)	0.8×0.8×2.2
**IV^5^**	71(69)	30,60	2000	34,48	240×240×(85,120)	2.5×2.5×2.5
**V^5^**	41(37)	30, 60	2000	34,48,49,50	240×240× (85–125)	2.5×2.5×2.5
		32,		34	240×240×85	2.5×2.5×2.5
**VI^6^**	113(107)	34,	1,2,3×10^3^	50	240×240×125	2.5×2.5×2.5
		50, 60		60	256×250×120	2.0×2.0×2.0
**VII^5^**	9(9)	70	1,2,3×10^3^	34	240×240×85	2.5×2.5×2.5
**VIII^5^**	20(20)	11,60	2000	34, 49 ,50	240×240× (85 – 125)	2.5×2.5×2.5
**IX**	1(1)	92	1600	50	240×240×125	2.5×2.5×2.5

1 Spatial dimensions are reported (left/right, back/front, foot/head).

2 Number of submitted datasets, parenthesis indicate number completed by pipeline.

3 Number of gradient directions (DWI volumes).

4 All data were collected using axial slices.

5 Contains within-study variation in some parameters. FOV changed with slice number to maintain consistent voxel size.

6 Contains within-study variation in some parameters. Voxel size and FOV changed with slice number as indicated.

The second group was chosen (i) for the formation of b_o_ atlases required for multi-atlas segmentation and (ii) to serve as a constant visual reference dataset for comparisons across QA reports. For this second group we use the DTI component of the open-access Multi-Modal MRI Reproducibility study [41]. Briefly, the study consists of 42 DTI datasets in total from 21 subjects, each scanned twice at 3T. Each dataset was acquired with a multi-slice, single-shot, echo planar imaging (EPI) sequence with 32 diffusion sensitizing gradient orientations at a b-value = 700 s/mm^2^ with five signal averages used for the minimally weighted volume. The resulting images consisted of 65 transverse slices with a field of view of 212×212 mm^2^, reconstructed to 256×256 in-plane matrix (voxel size 0.83×0.83×2.2 mm^3^).

### 3.2 QA Processing Pipeline

**Figure 2:**
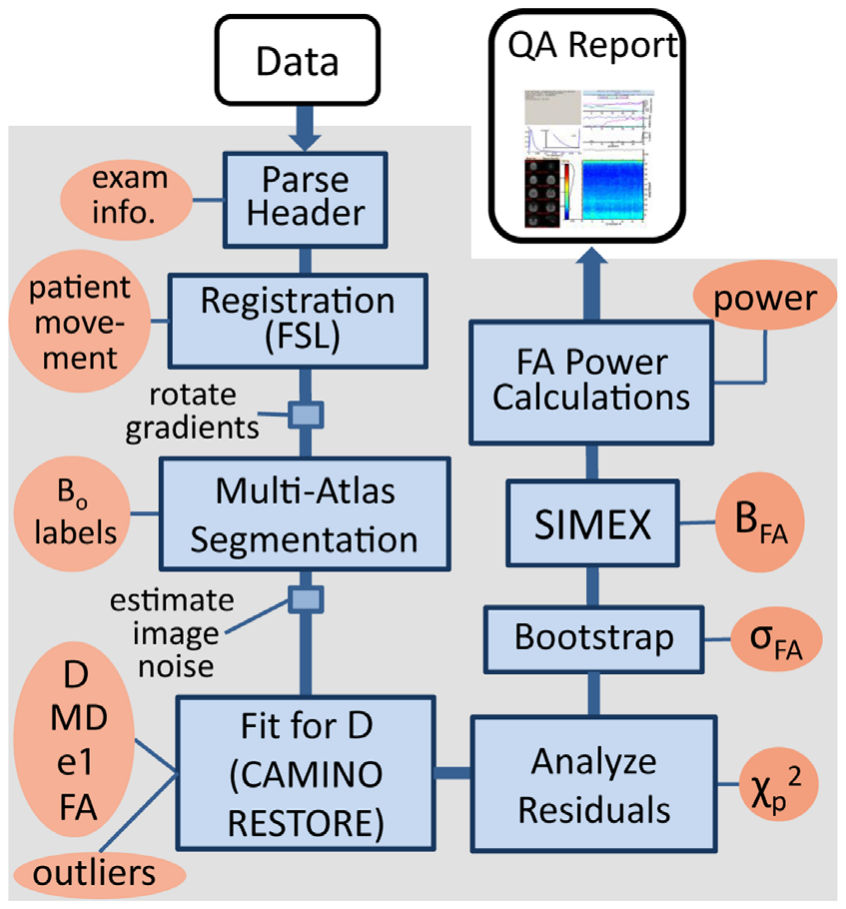
Flowchart of QA pipeline showing major processing steps (blue boxes) and outputs stored or graphed in the QA report (red ovals). Not shown is conversion of image data to NIfTI files (immediately after ‘Parse Header’), the creation of two brain masks after ‘rotate gradients’, one mask for CAMINO and a second slightly more restrictive mask for statistical analysis, and the implied additional calculation of 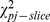 in ‘Analyze Residuals’.

**Figure 3:**
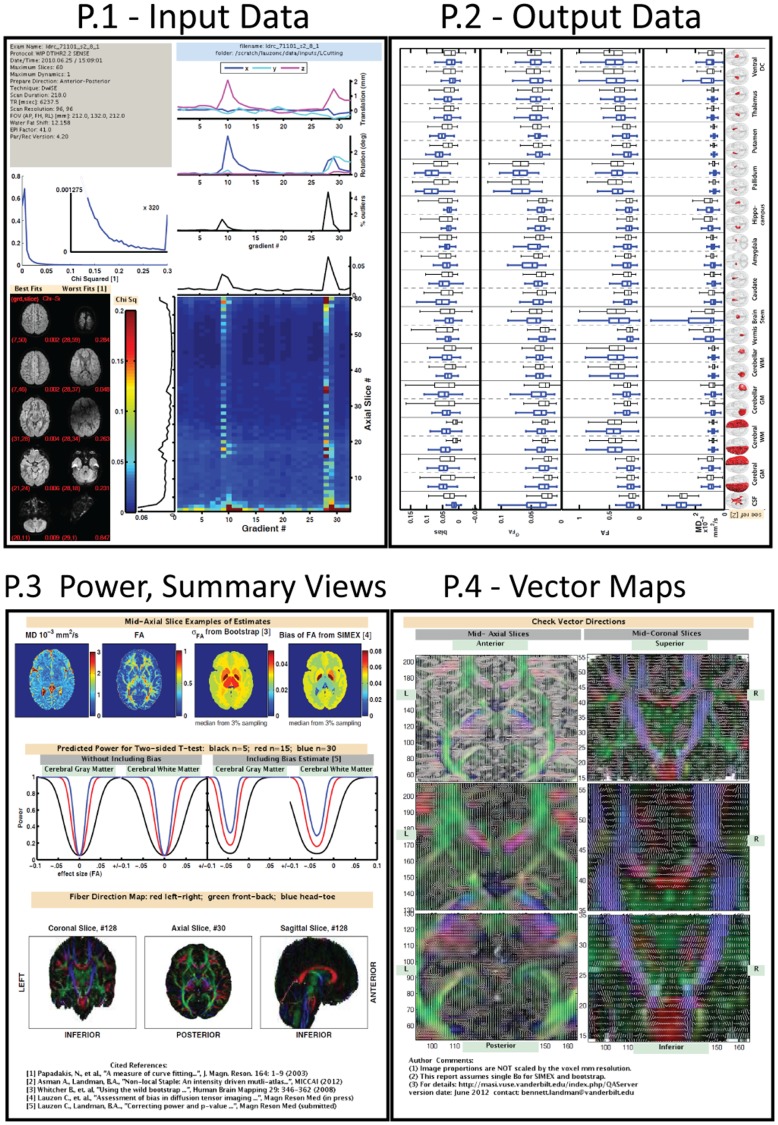
The four pages of the QA report. All data is shown from the same subject. The first page (P.1) parses the header (gray and blue boxes), plots patient movement in rotation and translation, plots RESTORE outliers per DWI (three upper right plots), plots 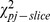 (color plot), shows five ‘best’ and ‘worst’ DWI slices (bottom left), and plots a histogram of 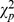 with an automatically determined magnification around the ‘noise’ lobe region as described in Papadakis et. al.. (P.2) shows a model of the 25 segmented regions (right column) and plots regional distributions of MD, FA, 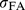, and *B*_FA_ (blue boxplots) adjacent to corresponding distributions from the Multi-modal dataset (black boxplots). (P.3) Page three displays mid-axial slice views for MD, FA,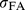, and *B*_FA_ (top row, left to right), select power curves for FA (middle column), and full mid coronal, axial, and sagittal slices for the vector colormap (R = right-left, G = anterior-posterior, B = foot-head). (P.4) Page 4 shows the vector directions (while lines, not clearly discernible at figure size) overlaid on the vector colormap for a mid-axial (left column) and mid-coronal (right column) slice. Three different enlargements are shown for improved viewing.

**Figure 4:**
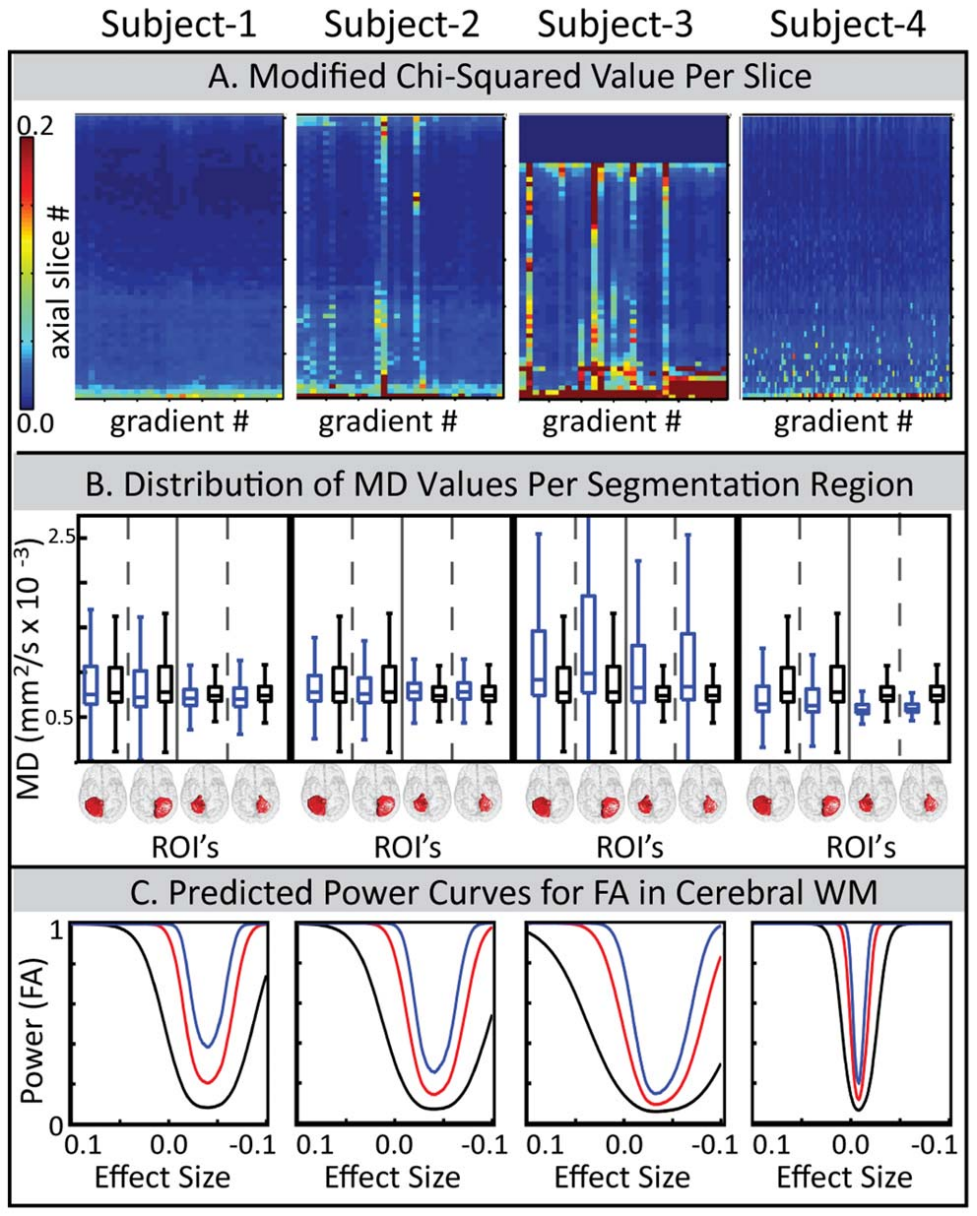
Comparing three pieces of the QA report for four subjects. Each piece is selected from a different page of the report. Subject 1 and subject 2 are from Study II and are the same subjects in Figure 1. Subject 3 is from Study I and subject 4 is from Study IX. (A) Colorplot of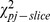. Within each colormap, one column represents an entire normalized diffusion weighted volume, and each row represents the same axial slice. High 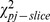 correspond to poor data with 0.2 being definitively bad. (B) MD distributions for four segmented regions, left cerebellar gray matter (GM), right cerebellar GM, left cerebellar white matter (WM), and right cerebellar WM. Blue boxplots indicate the distribution of each subject’s MD value while the black box-plot is the reference data from the multi-modal study. (C) Power curves for theoretical sample sizes of *n* = 5 (black), *n* = 15 (red), and *n* = 30 (blue). Curves include bias estimates and pertain to voxels in the cerebral WM.

Unless otherwise indicated, all processing and analysis was performed in Matlab 2010 (Mathworks, Natick, MA). External software was incorporated into the pipeline through Matlab system commands. Data processing was conducted using the resources of the Advanced Computing Center for Research and Education (Vanderbilt University, Nashville, TN). Data entering the QA pipeline is processed according to the flowchart in **Figure 2**. Blue boxes indicate processing steps and red ovals indicate outputs presented in a four page graphical QA report (**Figure 3**, **Figure 4**). Results presented on each page of the QA report and pipeline stored outputs are listed in **Table 2** and **Table 3**. To begin the pipeline, input Phillips par/rec data are imported into Matlab and converted to RAS (right-anterior-superior) oriented NIfTI image files using an open-source Matlab toolbox [44]. The program also accepts NIfTI inputs, but herein par/rec data is used. Conversion of all input data (including gradient tables) into an identical co-ordinate space greatly simplifies coding for the remainder of the pipeline and facilitated troubleshooting. The following section describes the pipeline roughly following data flow in **Figure 2**.

**Table 2:** Information presented on each page of the QA report.

Page 1	Page 2	Page 3	Page 4
Exam Info	Anatomical schematic of 25 segmented regions	Mid-axial slice visualization of MD and FA	Three mid-sagittal views of *e1* overlaid on vector color map with orientation labeling
Patient movement, translation and rotation	Boxplots of MD per region	Mid-axial slice visualization of median *σ*_FA_ and FA bias across segmented regions	Three mid-coronal views of *e1* overlaid on vector colormap with orientation labeling
Total voxels in each DWI scored as outliers by RESTORE	Boxplots of FA per region	Power curves with and without bias for gray and white matter cerebellum	
Histogram 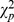	Boxplots of 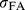 per region	Mid-axial view of vector colormap with orientation labeling	
Colormap of 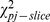	Boxplots of FA bias per region	Mid-sagittal view of vector colormap with orientation labeling	
Visual samples of best and worst DWI slices in each 1/5^th^ axial region of the brain. Best and worst are as graded by 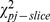	Boxplots of multi-modal dataset for each parameter.	Mid- coronal view of vector colormap with orientation labeling	

**Table 3:** Stored Pipeline Outputs.

DTI Processing	Statistical Analyses
registered data	segmented b_o_ volume
**D**	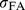
FA	*B*_FA_
MD	squared error per voxel
***e1***	power curves

#### 3.2.1 DTI processing to estimate the diffusion tensor

Upon data entering the pipeline, the par file (Philips parameter file – the data ‘header’) is parsed and important experimental properties are recorded. The DWIs are registered to the b_o_ in FSL (FLIRT, [28]) using a linear affine registration with 12 degrees of freedom for eddy current correction. The b_o_ volume is masked (FSL, BET, [27]) twice. One regular mask is used for DTI fitting, a second slightly more restrictive mask is saved for statistical analysis. Patient movement is measured by the registration matrix for each DWI and the gradient table is rotated accordingly.

Next, diffusion models are fit using the CAMINO software package [26]. To estimate the diffusion tensor a robust tensor fitting method is used (RESTORE, [25]). RESTORE requires an estimate of image noise as an input. For this estimate to be automatically calculated, we use an add-on module to the multi-atlas segmentation. Multi-atlas segmentation automatically segments the b_o_ into 25 regions. The standard deviation of signals across the segmented regions from each DWI volume is calculated and the median value is measured across the DWI, creating 25 estimates of standard deviation (one per segmented region). This estimate incorporates true noise and anatomical variability. The lowest noise estimate of the 25 could therefore be considered the closest to the true noise level, but to avoid the possibility of a regional artifact contaminating all DWI observations, the second smallest estimate of standard deviation is chosen as the estimate of noise. This method was tested with success on a small external subset of data with available repeat scans. CAMINO outputs the location of outlier voxels as determined by RESTORE, as well as the fitted diffusion tensor, and resulting tensor parameters of interest; FA, MD, and ***e1***.

#### 3.2.2 Multi-atlas segmentation of b_o_ volume

We employed a two-tier multi-atlas segmentation procedure in order to (1) create a collection of b_o_ atlases and (2) segment new b_o_ images using these newly created b_o_ atlases. The creation of the b_o_ atlases was performed using the multi-modal dataset which consisted of both T1-weighted and b_o_ images. Although the dataset consists of repeat scans, only one scan per subject was chosen. For each of the 21 subjects, the T1-weighted images were automatically labeled through multi-atlas segmentation using a collection 15 T1-weighted atlases from the Open Access Series of Imaging Studies (OASIS) dataset [43] that were previously labeled by an expert anatomist (courtesy of Neuromorphometrics, Inc., Somerville, MA). For each T1-weighted atlas, a collection of 26 labels (including background) were considered: ranging from large structures (e.g., cortical gray matter) to smaller deep brain structures. For each subject, all pairwise registrations were performed using ART [33] and the registered labels were then fused using Non-Local STAPLE [38]. Finally, a collection of 21 b_o_ atlases were then constructed by transferring the T1-weighted labels to the corresponding b_o_ images through an intra-subject rigid registration [45]. Using these newly created b_o_ atlases, the b_o_ volume from the DTI dataset are segmented through a similar multi-atlas registration procedure.

#### 3.2.3 Statistical Evaluation of DTI

Fitting errors and noise levels are evaluated through the pixel chi-squared. The fitted tensor output from CAMINO is projected back through the diffusion model (Eq. 1) to create fitted normalized DWI, **S_f_**. The original pixel-chi-squared 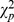 calculated (Eq. 3) and the histogram across spatial locations plotted in the QA report (**Figure 3** **,** page 1). The slice-pixel-chi-squared 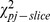
is calculated (Eq. 4) and the result per-slice per-normalized-DWI is plotted as a color image in the QA report (**Figure 4A**).

FA standard deviation and bias are estimated by bootstrap and SIMEX respectively. Both these methods are computationally expensive requiring many Monte-Carlo Simulations each. To keep the entire pipeline within a 24 hour processing period, linear least squares estimation is used rather than RESTORE, and only a sub-sampling of spatial locations undergoes bootstrap and SIMEX evaluation. Subsampling is performed using random selections across the brain volume. If necessary, continued random subsample per region is used until each brain region has at least 50 voxels sampled. The percentage of sub-sampling is indicated on the QA report, typically ∼ 4 % for larger datasets (e.g., 212×212×132 voxels) and ∼30 % for smaller datasets (e.g., 96×96×65 voxels). Bootstrap is performed as described in Theory 2.4 using 1000 Monte Carlo simulations. SIMEX is implemented as previously published [18]. Image noise parameter (Eq. 6) 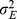 is estimated from the multi-atlas registration add-on module (Section 3.2.1). Data is corrupted with noise values *ω* = 2, 4, 6, and 8 using 2000, 4000, 6000, and 8000 Monte-Carlo iterations respectively for determining the expectation value of FA at each noise level. The trend in FA is fit with an order two polynomial and the unbiased estimate of FA determined from extrapolation to zero noise (ω = −1).

Power curves with and without bias are plotted for two regions, cerebral white matter (**Figure 4C**) and cerebral gray matter. Power for effect sizes ranging from −0.1 to 0.1 is determined at each voxel within each region using an analytic equation. The median power across the region for each effect size is recorded as the final power for that effect size. Note that only the subset of voxels for which 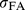
and *B*_FA_ were estimated can be included in the power analysis. Data necessary to create power curves for any other segmented region is stored by the pipeline (**Table** **3**).

### 3.3 QA Report Design

A graphical four page report is automatically produced by the pipeline to provide an easy to understand summary of the results. Layout design was therefore an important consideration of increasing the ease of using the QA report. The report is divided into four categorical pages (**Table** **2** **,** **Figure 3**). The first page of the report is designed to investigate quality of input data (b_o_, DWI, image protocol), the second page reports quality of output data (FA and MD), the third page includes summary axial-slice views of output data and power curves, and the fourth page displays the vector map images with the diffusion vector direction overlaid as a white vector across each voxel.

Several layout choices are worth noting. First the layout design on page one takes advantage of the observation that four of the variables are plotted with the DWI number (gradient number) on the abscissa. We therefore aligned the shared abscissa across these four graphs: chi-squared evaluation per axial slice, rejected outlier plot, rotational motion plots, and the translational motion plot. The visualization enables the eye to evaluate all four metrics simultaneously for each gradient direction. Second was the consideration of which output data to include graphically. Unlike input data which always consists of a gradient table, b-value, b_o_, and DWI, desired output data is study dependent and ‘unknown’ to an automatic pipeline. However, given the extra importance and unique insight output data quality may provide [46], we included quality analysis of typical scalar outputs of interest (FA and MD). We take advantage of the labeled brain regions made available through the multi-atlas segmentation method that used for the automatic noise estimation. Boxplots showing the distributions of FA, MD, 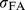
and *B*_FA_ are displayed *per segmented region* in order to have a greater chance of capturing unusual trends in the data. Additionally, to make comparisons easier across QA reports, the distributions from the 21 subjects in the multi-modal study are displayed and serve as a reference dataset. Third, the third page includes visualization of 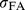 and *B*_FA_ . To obviate the problem of visualizing sub-sampled data, the median value per segmented region is chosen to represent 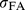 and *B*_FA_ for the entire region. Finally, the fourth page was included primarily as a self-checking mechanism to ensure proper spatial interpretation of gradients and images by the automatic pipeline. This feature will be particularly useful as pipeline use extends to a greater diversity of DTI datasets and DTI data storage structures.

### 3.4 Rubric Evaluation

#### 3.4.1 Experimental Design

A four question multiple choice rubric was used to evaluate the effectiveness of the QA report (**Table 4**). The rubric asked evaluators to assess DTI data quality. Comparison of novice responses to expert responses served as the metric for evaluating the effectiveness of the QA report. First, a total of fifty random datasets were selected from studies I, II, and III (the first three studies completed by the pipeline). Second, a pool of evaluators was established with one evaluator labeled ‘expert’ (2 years of experience with DTI data evaluation) and eight evaluators labeled ‘novice’ (minimal to no previous experience with DTI data). For each of the fifty studies, five rubrics were completed; (a) to create a ‘gold standard’ one of the five rubrics was completed by the expert using both image data and the QA report, (b) two of the five rubrics were completed independently by two randomly selected novices using the image data, and (c) two of the five rubrics were completed independently by the same novice pair using the QA report. Note each dataset is evaluated four times by two novices with each novice evaluating the same dataset twice in the opposite experimental order.

**Table 4:** Rubric Questions.

**Q1 (motion):** What is the overall range of motion?									
Very Small		Small	Moderate			Large		Very Large	
(∼0.25 mm)		(∼0.5 mm)	(∼1 mm)			(∼1.5 mm)		(∼2 mm)	
○		○	○			○		○	
**Q2 (gradients):** What number of gradients appear to be linked to generally poor-quality outlier images?									
None		1–2	3–5			6–10		+10	
○		○	○			○		○	
**Q3 (axial slices):** Divide the brain into four axial regions superior down to inferior. Excluding any axial slices at the inferior and superior periphery containing outliers across most gradients, estimate the number of slice outliers within each quadrant.									
Quadrant		0–5		6–10	11–25		26–50		+50
3a.- Superior		○		○	○		○		○
3b		○		○	○		○		○
3c		○		○	○		○		○
3d-Inferior		○		○	○		○		○
**Q4 (overall):** Given data as a while, select the overall evaluation and corresponding action you would take or recommend.									
	○ Unusually Excellent – Process and/or bring to attention of PI								
	○ Good – Proceed with processing								
	○ Acceptable – Proceed with processing								
	○ Poor – Bring to attention of PI, potentially proceed with processing								
	○ Unacceptable – Bring to attention of PI, recommend not using data and proceeding with alternative s (e.g., rescan patient, scanner evaluation, protocol evaluation).								

To control for the possible impact of learning, the order in which each novice completed the rubrics, both in terms of dataset numbers and whether a novice used the QA report or image data first, was systematically mixed and assigned. Additionally, a fixed minimum time width between an evaluators completion of the first rubric to the second rubric for the same dataset was set at one week. Users answered rubric questions through Google Documents which automatically recorded answers in a table that was imported into Matlab for evaluation.

#### 3.4.2 Statistical Evaluation

Rubric answers for novices using image data, novices using the QA report, and expert evaluation were analyzed to assess the effectiveness of the QA report. Five questions were evaluated: (1) Does learning impacting outcomes? (Evidence of learning may confound interpretation of results), (2) Does using the QA report impact consistency of different novices evaluating the same dataset? (3) Does use of the QA report significantly change the novice response to the rubric questions and overall assessment of quality? (4) Does use of the QA report impact novice errors/disagreement with an expert? (5) Does the QA report impact novice bias, e.g. are the errors centered around zero?

To answer these questions, summary statistics were calculated on the Rubric evaluations and several hypothesis tests were performed. Due to the non-normal distribution of the data, all hypothesis tests were non-parametric and robust (Wilcoxon rank sum and Wilcoxon signed rank). In addition to the mean and standard deviations of the responses, the following analyses were done. (1) For assessing learning a hypothesis test was conducted between (i) the pool of rubrics that were completed the first time for any given dataset (two per dataset) and (ii) the pool of rubrics that were completed the second time (two per dataset). (2) To evaluate the stability of novice quality evaluation, the inter-rater variability for each question was measured. Inter-rater variability was defined as the standard deviation across the differences of rubric answers corresponding to the same subject. The inter-rater variability was calculated for QA report based responses and image data based responses. (3) To detect a difference in overall assessment of quality, a paired hypothesis test was conducted between (i) rubric responses from novices using the QA report and (ii) rubric responses from novices using the image data, (4) to test for changes in errors, a hypothesis test was performed between (i) errors when novices used image data and (ii) errors when novices use the QA report. Finally, (5) bias was evaluated by using a hypothesis test to compare (i) errors from either responses using image data or QA report to (ii) a zero-mean distribution.

### 3.5 Low Dimensional Analysis of QA Metrics

**Figure 5:**
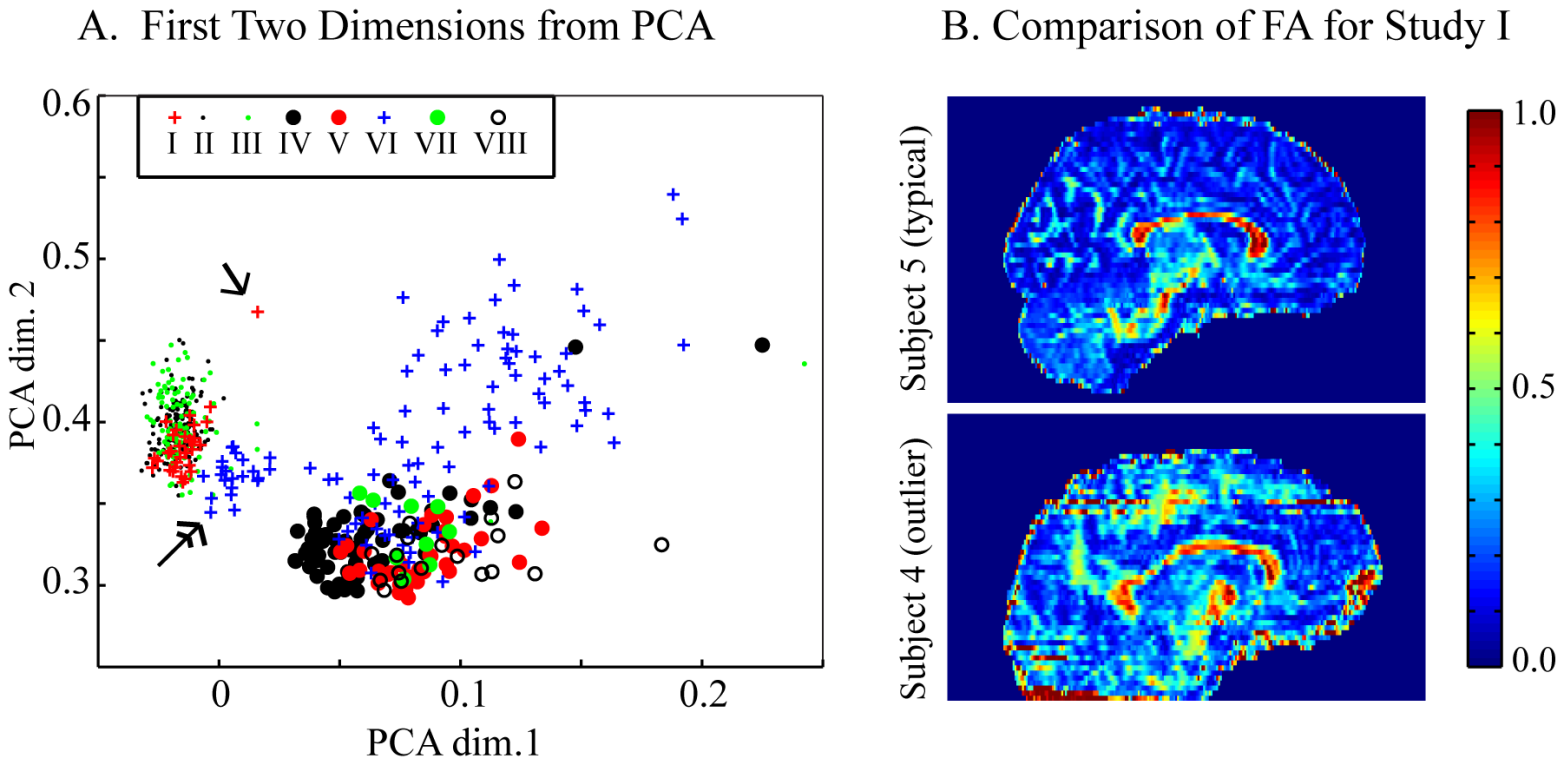
Statistical metrics stored by pipeline evaluated using PCA. Each of the 567 DTI datasets was characterized by a 112 element vector of stored outputs from the pipeline. PCA analysis was performed on the resulting data. (A) DTI dataset locations in the first two dimensions of the PCA analysis. Data is symbolized by study Roman numeral (**Table 1**). Single arrow points to a data quality outlier from study I; subject 3 in Figure 4 and in (B). A double headed arrow points to a cluster representing an isolated protocol sub-group from study VI. (B) FA maps from similar sagittal slice locations in two subjects from Study I. Subject 4 is the indicated outlier in (A) and subject 5 was selected from the center of the study I cluster seen in (A).

We evaluate if stored quality metrics from the pipeline capture important characteristics of DTI data through qualitative analysis of study clustering (**Figure 5**). The approach tests for a possible advantage of unifying the statistical pieces and offers one method for data exploration. In this effort, we also illustrate a possible approach that could be used in future efforts to more fully automate QA. This simple evaluation considers the data through the ‘bottom-line’ perspective of a clinical researcher, in which only impacts on final output data (rather than input) are considered [46]. First the regional boxplot distributions of MD, FA, 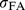, and *B*_FA_ are stored in a single vector for each DTI dataset. Note that this data captures elements of a power curve analysis as it includes the only two parameters needed for the power curve analysis, 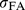, and *B*_FA_. To characterize data, the 25 segmented regions are first reduced to 14 regions through pooling left and right hemisphere regions where appropriate. For each region the mean and standard deviation of the boxplot data are recorded, creating a 112 element vector. PCA analysis across all DTI datasets is then used to reduce the data to its two most fundamental dimensions. To normalize variance measures between data of different scales (e.g., MD vs. 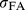), PCA is performed on z-scored data (mean subtracted, normalized by standard deviation). The resulting two-dimensional data is plotted and cluster behavior is qualitatively evaluated.

## Results

### 4.1 DTI Data and Pipeline Processing

To provide a relatively robust challenge for the pipeline, a total of 608 DTI datasets were submitted for processing. These datasets were arbitrarily selected from a large archive consisting of valid clinical research data, pilot scans, development scans, and scans that were incompletely acquired (i.e., terminated by the technologist during scanning). This highly heterogeneous dataset was chosen to ensure that the pipeline would complete for all datasets for which it was feasible to estimate tensors and return tractable error messages when datasets could not be processed. The datasets ranged in protocol (**Table 1**), quality, and contained naturally occurring examples of the difficulty of assessing DTI data quality. **Figure 1** shows subsample images from similar anatomical axial slices of two study II datasets. These datasets are labeled subject 1 and subject 2. The DWI and b_o_ of both data appear of similar quality with no clear artifacts, yet a subtle anomalous region of high FA values can be seen in the middle right (patient right = image right) hemisphere of subject 2. The question arises regarding if the anomaly is real (i.e., a tumor) or the product of poor data quality not manifested as a clear artifact.

The pipeline handled well the challenge of processing diverse DTI data. Of the 608 submitted datasets 568 datasets completed the full pipeline processing. One dataset was excluded after successful processing because the report showed severe issues with limited field of view and motion artifacts (i.e., an incomplete dataset). The datasets that did not successfully complete processing were individually inspected. All failures were due to corrupt data of one of three types: (i) par file errors such as a missing gradient table, (ii) data structure errors (e.g., missing slices), and (iii) datasets not containing a b_o_ because they were part of a larger multi-b-value study.

Total processing time for each dataset was targeted to be approximately 24 hours of total CPU time. Longer turn-around times diminish the ability of researchers to respond to poor data through experimental methods (including patient re-scans) and attenuate the benefits of automatic methodology. From test cases, time consuming pipeline steps included registration (∼ 20 minutes) and multi-atlas registration (1 – 9 hours). The most time consuming steps however are bootstrap and SIMEX. Long turn-around times are needed for bootstrap and SIMEX because they rely on Monte-Carlo simulation which is inherently improved through greater repetition numbers. Average processing time across the 567 datasets was 22.2 hours with a range (14.8, 27.8). Total processing time was 525.2 days, which was reduced to 3 days through cluster parallel processing. Note that the average reported herein (22.2 hours) is for a single node. The program itself does not use parallelization, and cluster analysis was only used to process 608 datasets simultaneously.

### 4.2 Four Page QA Report

It is not possible to present all results or observations regarding the QA report. We therefore highlight a small subset of particularly informative pieces and example how these pieces may be interpreted by a researcher using DTI QA. Three pieces of the QA report are compared for four different DTI datasets (**Figure 4** **).** Subject 1 and 2 are from study II and are the same subjects depicted in **Figure 1** **.** Subject 3 is from study I and subject 4 is an informative ‘out-of-study’ comparison from study IX. The presence of subject 4 is primarily to enable a positive control for the pipeline since it is already known that larger datasets (see protocols in **Table 1**) should produce higher quality data.

First, we consider the response of the pixel chi-squared value calculated per slice (Eq. 4, **Figure** **4A**). This metric was plotted on a color graph with a fixed range of 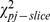 = [0 0.2], where 0.2 is definitively poor quality [22]. Subjects 1, 2, and 3 show decreasing data quality as indicated by the increasing number of poor outlier axial slices in each subject. Note that poor axial slice outliers tend to align along the same gradient direction, as indicated by the vertical striping patterns seen in subjects 2 and 3. This observation was common and, in many cases, linked to large patient motion for that gradient direction (data not enlarged but seen in **Figure 3**, page 1). The last dataset contains 92 diffusion sensitizing gradient directions, roughly three times as many as studies 1 and 2. For this subject, random poor axial slice outliers can be seen clustered at the lowest axial slices. This was common across QA reports and expected given this region corresponds to anatomical regions of low signal (e.g., brain stem). The rectangular region of blue seen in the top of the 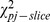 plot for subject 3 indicates the slices were outside the anatomical regions of the brain. The rectangular blue pattern was also common among the QA reports.

Next, we consider the boxplots from page 2. Specifically, **Figure 4B** examines the response of the boxplot distributions of MD for four segmented regions: the left hemisphere cerebellar gray matter, the right hemisphere cerebellar gray matter, the left hemisphere cerebellar white matter, and the right hemisphere cerebellar white matter. The thick dark blue boxplot indicates the spread of MD values for the processed dataset, and the thin black boxplot is the spread of values from the multi-modal study. Although 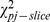 was higher for subject 2, the boxplots for subject 2 appear to be of comparable (perhaps superior) quality than subject 1, suggesting that the lower quality DWI for subject 2 may not have impacted this region and/or the MD results. However, the MD boxplots for subject 3 clearly demonstrate an increase in spread. This change was universal across all MD values for subject 3, indicating this dataset is a significant poor quality outlier to other subjects in the study.

Finally, we investigate how data quality is manifested in the power curves. **Figure 4C** shows the plots of the power curves for the cerebral white matter (CWM), shown for *n* = 5 (black), *n* = 15 (red), and *n* = 30 (blue). These power curves include incorporation of the bias estimate (Eq. 7). An unbiased power-curve would have its minimum value at ES = 0. The bias in subjects 1–3 is apparent in the shift in their power curves away from zero. In all three subjects, the median bias for the CWM can be seen by the power curve to be approximately +0.04. Subject 4 has a minimally biased power curve, which is expected from its much larger gradient table. The width of the power curves indicates the overall power, with broader curves being less powerful. Thus, the curves narrow as sample size increases. The curves also broaden across subjects 1, 2, and 3, indicating a decreasing quality across these three subjects and trending with the 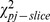 graph.

### 4.3 Rubric Results

An important question is if the chosen visual representation of the QA report conveys important statistical information provided by the pipeline and improves overall human quality evaluation accuracy. Comparison of rubric answers from novices using either image data or the QA report to expert answers enabled a controlled evaluation of quality analysis accuracy provided by the QA report. Summary metrics of rubric responses are provided in **Table 5**, results of hypothesis tests are provided in **Table 6**, and an analysis of man-hours needed for evaluation are in **Table 7**.

**Table 5:** Summary Metrics for Rubric Evaluations.

	Mean^1^ (Expert)	Mean^1^ (Images)	Mean^1^ (Report)	Inter-rater^2^ variability (Images)	Inter-rater^2^ variability (Report)	Mean^3^ Errors (Images)	Mean^3^ Errors (Report)
**1. Motion (mm)**	0.8 ± 0.5	1.0 ± 0.4	0.9 ± 0.5	0.8	0.4	0.5	0.3
**2. Gradients**	1.1 ± 2.0	2.1 ± 2.0	1.7 ± 1.7	3.1	2.6	1.5	1.2
**3a. Axial Slices**	5.8 ± 6.0	15.9 ± 12.0	9.9 ± 9.6	23.0	17.9	11.3	5.9
**3b. Axial Slices**	3.7 ± 3.8	12.9 ± 9.2	6.5 ± 6.2	16.7	12.3	9.3	3.5
**3c. Axial Slices**	5.3 ± 7.8	15.5 ± 9.7	9.9 ± 8.9	18.5	19.9	10.6	6.7
**3d. Axial Slices**	9.7 ± 12.2	21.5 ± 11.1	20.2 ± 11.6	28.2	27.6	16.9	14.3
**4. Evaluation** ‡	2.7 ± 1.0	2.1 ± 0.7	2.6 ± 0.0	0.9	0.8	0.8	0.4

1 Mean reported as mean +/- standard deviation. Are the rubric responses significantly different for Report vs Images?

2 Inter-rater variability is calculated as defined in Methods text.

3 Mean calculated on magnitude of the errors.

‡ Evaluation responses were assigned values 0 through 5, with 0 being ‘Unacceptable’ and 5 being ‘Unusually Excellent’.

**Table 6:** Rubric Evaluation Using Non-parametric Hypothesis Testing.

	Q1. Responses^1^	Q2. Errors^2^	Q3. Image^3^	Q4. Report^4^
**1. Motion**	p = 0.08	p < 0.001	p < 10^−4^	p < 0.01
**2. Gradients**	p < 0.05	p = 0.35	p < 10^−5^	p < 0.01
**3a. Axial Slices**	p < 0.01	p < 0.01	p < 10^−7^	p < 0.05
**3b. Axial Slices**	p < 0.01	p < 0.01	p < 0.01	p < 0.001
**3c. Axial Slices**	p < 0.001	p < 0.01	p < 0.01	p < 0.001
**3d. Axial Slices**	p = 0.61	p = 0.06	p < 10^−4^	p < 0.01
**4. Evaluation** ‡	p < 0.001	p < 0.001	p < 10^−7^	p = 0.43

1 Q1. Are the rubric responses significantly different for Report vs Images?

2 Q2. Are the rubric errors significantly different for Report vs Images?

3 Q3. Are the rubric errors significantly different for Raw vs Zero mean distribution?

4 Q4. Are the rubric errors significantly different for Images vs Zero mean distribution?

‡ Evaluation responses were assigned values 0 through 5, with 0 being ‘Unacceptable’ and 5 being ‘Unusually Excellent’.

**Table 7:** Time Evaluation.

time (min)^1^	Expert	Report	Raw
mean +/− std	2.2 ± 1.3	1.9 ± 0.9	6.9 ± 2.9
(min, max)	(0.7, 5.7)	(0.7, 5.9)	(1.0, 24.8)

1 The time difference between Report and Raw are statistically significant (p < 0.001).

First evidence of learning was evaluated as it may confound interpretation of the novice responses (statistical results not included in **Table 6**). No evidence of learning was found between the first and second evaluations (p > 0.05). However when only responses using image data were considered, there was evidence of learning in the evaluation of motion. Reported motion decreased by a significant amount (p < 0.05 for the second evaluation. No significant differences were detected when only responses using the QA report were considered. Because learning was overall not significant, the rest of analysis was not complicated by accounting for possible learning.

Novice responses were found to be significantly different for the majority of rubric questions when novice’s used the QA report versus when novice’s used image data (**Table 6**, Q1). Use of the QA report increased the similarity between the average expert evaluation across all 50 subjects and the novice evaluation. The QA report generally lowered the inter-rater variability and universally decreased the magnitude of novice errors (**Table 5**). The decrease in error was found to be statistically significant for most questions. QA report and image data yielded biased evaluations for all rubric questions except the ‘overall evaluation’ which has no evidence of bias when the QA report was used (**Table 6**, Q4, p = 0.43) and a strong evidence of bias with image data (**Table 6**, Q4, p < 10^−7^).

Another significant factor when evaluating the effectiveness of the QA report is human hours saved through its use. Use of the QA report saved a statistically significant amount of time. On average the QA report saved the novice 70% of total time while maximum time spent on any one dataset was reduced from 25 minutes to 6 minutes (**Table 7**). Overall, the results indicate the use of the report improves the accuracy of quality analysis evaluation and saves a large and statistically significant amount of human effort.

### 4.4 Low Dimensional Analysis of QA Metrics

PCA reveals that the regional statistical properties of MD, FA, 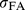 , and *B*_FA_, provide important characteristic information on the DTI datasets. Although intra-study protocols are variable for many of the studies (**Table 1**), in the reduced two dimensions, data is seen to cluster according to study number (**Figure 5A**). In particular studies I – III cluster closely followed by studies IV, V, and VII. Study VI is the study with the most diverse protocol range and correspondingly contains the least tightly clustered datasets. Interestingly, a sub-cluster from Study VI is clearly seen adjacent to the studies I – III cluster (**Figure 5A**, double headed arrow). This sub-cluster completely and exclusively includes study VI data of identical protocol (b-value = 1000, 32 diffusion sensitizing gradient directions, 60 slices).

The observation that data clusters by study may seem trivial, but the clustering is important. Because data of similar protocol groups together, it then becomes reasonable to conclude that study cluster outliers may correspond to data of poor quality and differing metric behavior. This is seen to be the case in investigated outliers, for example, the study I red cross outlier (**Figure 5A**, single headed arrow), belongs to the poor quality dataset of subject 3 in **Figure 4** and **Figure 5B**. Although these clusters are only shown for the first two dimensions of PCA, these dimensions account for the majority of the variance. Additionally, inclusion of the third dimension, which explains an important portion of the variability, did not affect the clustering trends observable in the first two dimensions (data not shown).

## Discussion

QA in scientific data is important, but is particularly crucial for DTI experiments which challenge hardware and gradients, induce unique motion artifacts, and require patient cooperation for multi-volume acquisitions. Manual QA on the raw data is not only tedious but difficult since poor data does not always manifest as clear artifacts and artifacts themselves can mask as natural variability between volumes (**Figure 1**). If significant, poor data can alter important diffusion measurements (such as FA in **Figure 1**) which in turn may present as seemingly true anatomical variability between subjects. Even if artifacts were more clearly present to the human eye in the DTI data, a concern arises regarding the number of human hours required to detect that artifact within the context of a study (such as 183 datasets for the anomaly in **Figure 1**). Artifact detection software (DTIPrep) facilitates artifact detection, but cannot give warning to non-artifact based quality analysis problems and still requires human intervention for each DTI dataset. Because of these time consuming hindrances, the question arises of whether the anomaly might even go unnoticed under general practice.

To improve QA, we have combined in-situ pipeline methodology with statistical analyses that have been previously identified as key components of DTI QA. These data are saved and important components graphically represented in a four page automatically generated report. The pipeline itself successfully processed diverse DTI data (**Table 1**) and revealed interesting trends. It was not uncommon, for example, for patient motion to correlate with poor fitting DWI (**Figure 3** **,** page one). In terms of correlation between quality of input data and quality of output data, note that CAMINO RESTORE uses robust fitting algorithms, and by design output data (e.g., FA) is less sensitive to artifacts present in input data. However, trends were still observable. Quality of input data trended with the regional distributions of output data in the extremes of quality, as seen in subjects 1 and 2 compared to subject 3 of **Figure 4B**. The power curves demonstrated sensitivity to input data quality, but their interpretation appears nuanced and study specific. For example, the power curve for subject 1 in **Figure 4C** indicates good quality for study I, but clearly not for study IX (subject 4). The power curves and regional output distributions will likely be especially informative when combined with the pipeline stored power curves and regional output distributions from other subjects within the same study. The easy ability to make such observations about inter-protocol quality is an additional benefit and potential secondary use of the QA pipeline.

One objective of the pipeline was to evaluate the QA report’s ability to save human hours and improve QA. We demonstrate the report improves QA even when an individual has the ability/time to inspect all acquired data. The novices provided distinctly different responses with and without the QA report, as indicated by the hypothesis test (**Table 6** **, Q1**). The novice responses using the QA report had greater similarity with the experienced researcher responses (**Table 5**). Perhaps most importantly, use of the QA report reduced the errors and eliminated the bias of a novice’s overall evaluation of data quality (**Table 6**, bottom row).The time saved doing QA using the report was statistically significant and quite substantial, particularly when considering QA on multiple DTI datasets as might be required for evaluation of data for a complete study.

The pipeline offers clinical researchers easy access to modern statistical methodology tailored for DTI while simultaneously performing essential pre-processing and model fitting. The pipeline stores analysis outputs tailored for more detailed investigation/follow-up. Many possibilities exist for developing approaches that use the stored outputs to evaluate protocol differences, cohort differences, and data quality outliers. Herein, we demonstrate one of many possible approaches and explore the sensitivity of the metrics to DTI data characteristics. The metrics were found to be sensitive to DTI protocol type with similar protocols clustering together in two-dimensional PCA. Because DTI data clusters according to protocol, within-protocol cluster outliers become poor quality suspects, as was the demonstrated case for an outlier from study I. This outlier detection approach suggests one possible methodical pathway for summarily evaluating statistical compatibility and classifying data as ‘usable’ or ‘unusable’. Although interesting for future investigation, the primary focus of this paper is on DTI processing and QA rather than machine learning, so we leave optimization/characterization of fully automated QA for future work.

Herein, our goals were to present the pipeline, demonstrate the visualization method succeeds in aiding QA, and provide data that demonstrates an advantage of unifying the statistical pieces. Yet an important question is ‘what decision should a scientist be making given the data in the pipeline?’. As the pipeline is the first time many of these statistics have been made readily available for scientists, the meaning of the individual pieces and their combined interpretation remains a large space for exploration. In the article, pieces of the QA report have been highlighted for guidance on interpretation (Section 4.2) and one method for data exploration that combines multiple pieces of the stored output has been presented (Section 4.4). However, we try to communicate these pieces without limiting the possibilities to the scope presented in the article. In regards to ‘what should a scientist do’, the answer depends upon the scientist’s needs, the results of the QA report, and the community’s growing understanding of the meaning of many of these metrics; an understanding that can be facilitated by multi-metric processing pipelines such as the one presented herein.

## Conclusion

Integration of processing software and isolated statistical metrics into a single automated QA pipeline significantly improved QA for DTI data. The presented pipeline offers the benefits of (1) an in-situ analysis option when integrated with MRI scanners (as currently running at a local facility), (2) encourages deeper data analysis and exploration by making existing state-of-the-art computational and statistical methods readily available to clinical researchers, and (3) provides a summary PDF of QA metrics to enable timely (within approximately 24 hours of data collection) quality analysis evaluation, enabling early experimental response to poor data quality and improved DTI methodology. The four page QA report was demonstrated effective in improving quality evaluation of DTI data, both in terms of accuracy and in time saved. The pipeline outputs, as demonstrated herein, can then be used outside of the graphical representations in the QA report for further analysis of data characteristics.

The analysis software is available in open-source under the Lesser GNU Public License (LGPL) 2.1+ at http://www.nitrc.org/projects/masimatlab. Please see the “mediawiki” section for download instructions and documentation on the “DTI QA Pipeline” sub-project.
